# Modelling the Geographical Origin of Rice Cultivation in Asia Using the Rice Archaeological Database

**DOI:** 10.1371/journal.pone.0137024

**Published:** 2015-09-01

**Authors:** Fabio Silva, Chris J. Stevens, Alison Weisskopf, Cristina Castillo, Ling Qin, Andrew Bevan, Dorian Q. Fuller

**Affiliations:** 1 University College London, Institute of Archaeology, London, United Kingdom; 2 Peking University, School of Archaeology and Museology, Beijing, China; University College Dublin, IRELAND

## Abstract

We have compiled an extensive database of archaeological evidence for rice across Asia, including 400 sites from mainland East Asia, Southeast Asia and South Asia. This dataset is used to compare several models for the geographical origins of rice cultivation and infer the most likely region(s) for its origins and subsequent outward diffusion. The approach is based on regression modelling wherein goodness of fit is obtained from power law quantile regressions of the archaeologically inferred age versus a least-cost distance from the putative origin(s). The Fast Marching method is used to estimate the least-cost distances based on simple geographical features. The origin region that best fits the archaeobotanical data is also compared to other hypothetical geographical origins derived from the literature, including from genetics, archaeology and historical linguistics. The model that best fits all available archaeological evidence is a dual origin model with two centres for the cultivation and dispersal of rice focused on the Middle Yangtze and the Lower Yangtze valleys.

## Introduction

Rice is one of the major world crops, and more than any other, has supported dense human populations and state systems in eastern, southern and southeast Asia through much of history and later prehistory (e.g. [1–3]). The origins and spread of cultivated rice has therefore been a major research theme in the archaeology of China, Southeast Asia and India, as well as amongst rice scientists working on genetic diversity (e.g. [4–8]). Approaches to locating origins have included inferences from modern genetics (e.g. [9,10]), from the occurrence of phytoliths in ocean cores (e.g. [11]), from phytoliths in archaeological sites (e.g. [12,13]), and from the presence of charred grains in archaeological excavations or rice husks in early pottery [13,14]. Methodological issues surrounding how to distinguish between archaeobotanical evidence for and model the evolutionary separation of wild gathered rice, pre-domestication cultivation and domestication processes have been increasingly addressed in recent years (e.g. [15–19]). Recent research has distinguished a phase of pre-domestication cultivation, lasting two to three thousand years, preceding full domestication [20,21]. Such discussions have tended to highlight a protracted process of domestication that was completed in the middle Holocene between 6000 and 3000 BC, suggesting that dispersal processes would have begun during or after this period. The origins and spread of cultivated rice has been implicated in the spread of human population groups and the distribution patterns of most major language families in the region, but again there has been little agreement about the source region(s) (e.g. [22–34]). While pinning down precisely when and where rice was first brought under cultivation, and how this relates to domestication as a subsequent extended process is an ongoing challenge [20,21], the archaeobotanical evidence for rice under both pre-domestication cultivation and subsequent to domestication is extensive enough that it warrants a systematic analysis in terms of which hypotheses of geographical origins are more or less reasonable. Therefore, in this paper, the term cultivation refers both to pre-domestication cultivation and farming of crops.

In the present paper we present the results of a modelling effort that calculated the most probable areas for the origins of the dispersal of cultivated rice by comparing models of spread from all possible points within the rice-growing domain. We assume simple least-cost distances which are then fit to known occurrences of rice in time and space. This in essence uses the existing knowledge of archaeological evidence of rice to infer backwards towards probable areas of origin. It also allows us to calculate the goodness-of-fit of various published hypotheses of the region of rice origin to the overall archaeological rice dataset. Although the distribution of archaeological rice evidence is uneven, with some areas having little evidence, there are enough data across a wide enough region to constrain a model of dispersal and provide a measure of the statistical likelihood of different potential origins. Therefore in addition to arriving at a most probable centre of origin from an unconstrained search of the rice growing domain and the archaeological evidence, we also test seven literature based hypotheses for region(s) of origin and quantify how well these fit the archaeological dataset. Finally, we take a closer look at the best-fitting model in order to identify outliers—archaeological sites that are earlier than predicted by the model—as these can identify regions where an additional origin or more complex geographical dynamics are possible and require further archaeobotanical investigation.

## Materials

**Fig 1:**
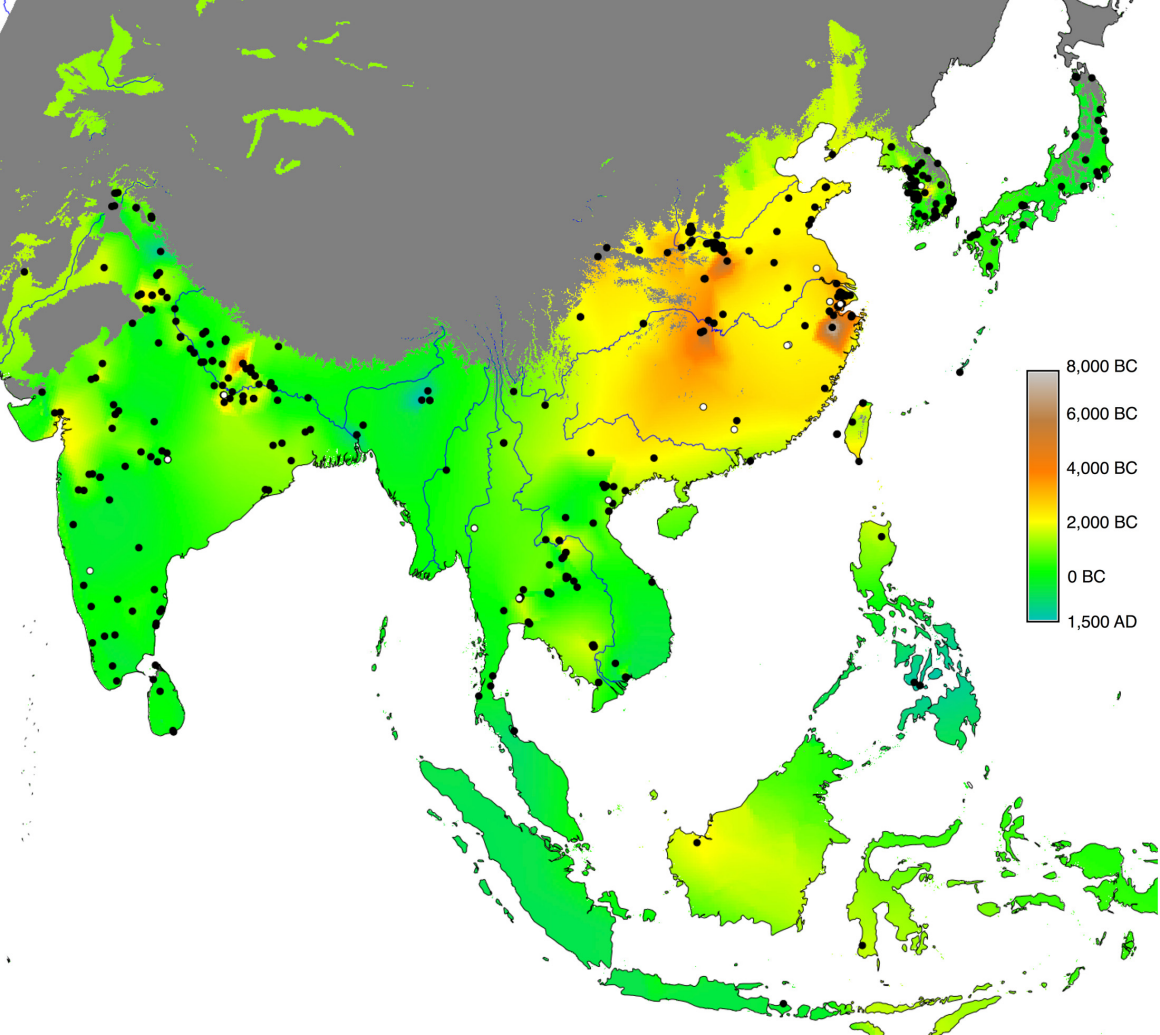
Map of the Rice Archaeological Database version 2.0. Sites with cultivated rice are shown as filled black dots, whereas open ones represent archaeological sites with wild rice. The background layer shows the quantile interpolation of the median dates for the arrival of cultivated rice. The grey-shaded region has been excluded from the interpolation (see main text). Major rivers have been included for reference (source: ESRI World Major Rivers).

Our empirical evidence is an updated Rice Archaeological Database (version 2.0, S1 Map). The first version of this database was used for a synthesis of rice dispersal by Fuller et al [35], while a slightly expanded dataset (version 1.1) was then used to model the dispersal of rice, land area under wet rice cultivation and associated methane emissions from 5000–1000 BP [36]. The database records sites and chronological phases within sites where rice has been recorded, including whether rice was identified from plant macro-remains, phytoliths or impressions in ceramics. Ages are recorded as the start and end date of each phase and a median age of the phase is then used for analysis. Dating is based on radiocarbon evidence, conventional or AMS, where available (220 phases) and based on a cultural association elsewhere. In addition to recording the presence of rice, evidence for domesticated or wild status is recorded as well as an inference of whether this was wet or dry cultivation ecologies of rice [37]. Version 1.1 included information on 385 sites, with 457 phases (some sites have multiple phases). Version 2.0, used in the current analysis, includes 400 sites and a total of 470 phases. The spatial and temporal distribution of the database can be seen in Fig 1 below, where the sites with rice that is either domesticated or on the path to domestication, are mapped as filled black dots, and archaeological sites with wild rice are mapped as open dots. The coloured background layer corresponds to a quantile regression and interpolation of the median dates of each site phase with cultivated rice, giving greatest weight to the earliest 10% of dates (see pp. 748–749 and supplementary information of [36] for details on this methodology). It therefore illustrates the earliest attested archaeological dates of cultivated rice throughout eastern, southern and southeast Asia.

## Methods

Simple mapping of the earliest dates for rice for any given region, such as that in Fig 1, can be quite informative. However, such approaches are limited by sampling issues and critics can point out, not incorrectly, that poorly sampled regions could in the future yield dates older than those already identified. This is particularly true for interpolation since gaps in the data are filled based merely on nearby sites and not on the entirety of available data. Alternatively, regression models have been used to identify the source(s) of dispersal events, estimate rates of spread, identify outliers and fill in sampling gaps with recourse to the entire dataset (see various references below). This is the approach taken in this paper.

Regression modelling requires two quantities for each data-point: an estimate of age and an estimate of distance travelled. Radiometric determinations can be, and have been, readily used as estimates of age for such cases (see examples below). However, the actual travelled distance is not so readily obtained as one cannot know exactly the path-history between the dispersal origin and the archaeological site. One can, nevertheless, estimate it. Geodesic distances are the shortest possible distances between two points on the surface of the earth, commonly known as “as-the-crow-flies” distance or great circle distances. They are easy to calculate and this approach has been taken by several scholars (eg [38–40]). However, they grossly underestimate the actual distances travelled as geodesics are blind to obstacles to movement [41]. Whereas actual people have to negotiate their movement around obstacles, further adding to the distances travelled, geodesics assume that people travel in straight lines—as straight as they can be on the surface of the Earth—and through possible obstacles, such as insurmountable mountains or a sea.

One very common alternative is to estimate distances in a more realistic landscape where there can be features that incur differing costs of moving ([42–45], to name but a few). One can then retrieve the distance travelled along a computational shortest path given the above landscape costs (hereafter referred to as a cost distance, for short). However this approach involves the need to postulate *a priori* a friction surface—a raster where each grid cell has a value, the local friction factor that affects the local rate of propagation. Thus one can postulate high-speed corridors and low (or null) speed barriers which will need to be negotiated. Friction values would also need to be either postulated or independently estimated, for instance via an exhaustive parameter space search, an optimization algorithm such as a Genetic Algorithm, or other parameter estimation methods (eg. [41,46,47]). Friction surfaces are then models in the truest meaning of the word: they are simplifications of relevant aspects of a real world situation. Like any other model their reliability depends on the importance of the aspects chosen as relevant, as well as on their parameters. The simplest cost distance model is a near-geography-free one where unsurpassable obstacles—such as the sea—and regions without archaeological evidence are masked out of the modelling. This forces the dispersing wave-front to negotiate travel around these regions, making it a step up from geodesic distance (which is completely geography-free, as argued above). Under these simple modelling assumptions, islands are not reached unless some form of coastal transport or sea-bridge is also included. This is the modelling framework which we have adopted in this paper, leaving alternative distance measures, such as those based on circuit theory (eg [48,49]), as well as the exploration of the role of geographical features (eg [41]) and a discussion of the nature of the dispersal (demic vs cultural) for future works.

Cost distance modelling of this kind was done using the Fast Marching algorithm developed by Sethian [50] and adapted for this purpose by Silva and Steele [41,51]. This method computes the cost distance of an expanding front at each point of a discrete lattice or raster from the source of the diffusion. It is flexible enough to allow for scenarios with different competing diffusive processes [46,51] as well as realistic heterogeneous surfaces [41,47].

**Fig 2:**
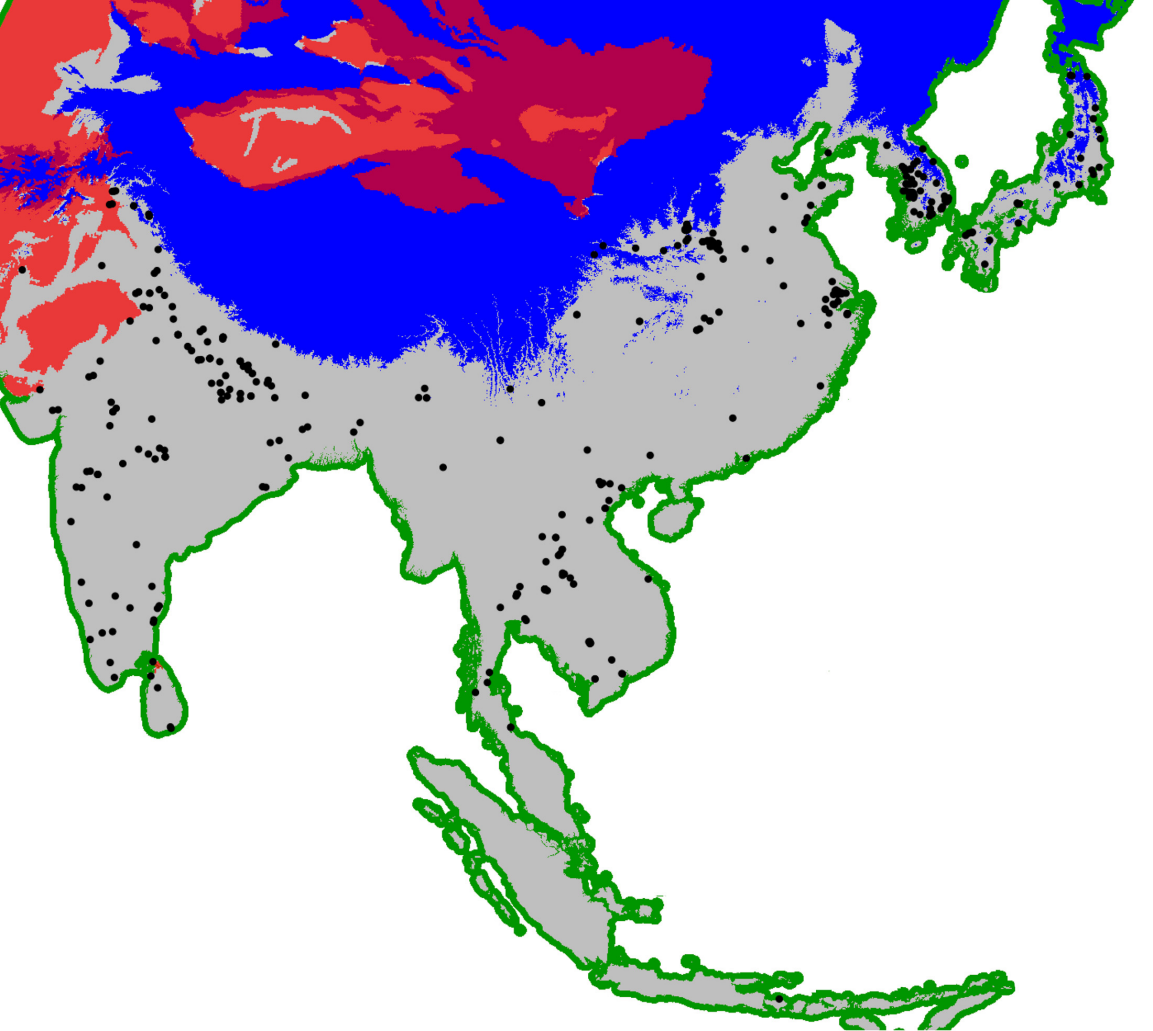
Domain used for the modelling. Regions excluded were those with annual total number of degree-days lower than 2,500 (blue-shaded) and desert eco-regions (red-shaded). Kept were the landmass region (grey-shaded) and 40 km offshore buffers for coastal transport (green shaded). Database sites with cultivated rice retained for analysis marked as black dots.

In this paper we have implemented a near-geography-free scenario where the available domain is restricted by two ecological factors: firstly, regions with a total number of degree-days in the year (a function of temperature important for crop growing see eg. [52–54]), lower than 2,500 were masked out since rice cannot grow in these regions and very little archaeobotanical evidence for rice was found in these regions; secondly, desert regions, where rice also cannot grow, were masked out. Degree-days were computed based on mid Holocene monthly temperatures retrieved from the WorldClim website [55]. Desert eco-regions were obtained from the freely available Terrestrial Ecorregions of the World dataset [56]. All of these were converted to a Lambert Azimuthal Equal Area projection that preserves the area of each cell in the domain. Coastal transport was also included by buffering out 40 km off-coast. This ensures that archaeobotanical data of key islands, such as the Japanese archipelago, are included in the analyses as it creates maximum 80 km offshore bridges connecting them to the mainland. Sadly, this minimalist approach to maritime transportation excludes data from Taiwan and other Southeast Asian islands that are more than 80 km distant from each other or the mainland. This issue will be addressed in a future paper looking at the dispersal routes of rice farming. The Rice Archaeological Database contains 330 records corresponding to the oldest evidence of rice domesticated or on the path to domestication that fall within this domain and were thus retained for the analysis in this paper. These are shown in Fig 2.

### Non-linear dispersal processes and Quantile Regression

Although the dispersals associated with different prehistoric innovations are often modelled as constant speed processes, this assumption is not necessarily always valid (eg see the discussion in [41]). The present Rice Archaeological Database includes not only sites with fully domesticated rice, but also older sites with rice that is clearly undergoing selection for domestication. As argued elsewhere domestication is a long process, taking two to three thousand years to complete (eg [16,20]). During this period, it is unlikely that rice cultivation would have spread that far beyond its centre(s) of innovation, and indeed there is little to no evidence for this. Rather, one would expect its dispersal to start very slowly, but quickly pick up pace once rice nears full domestication. Fort et al [39] had also noticed a similar feature for the European Neolithic but they opted to remove data prior to the Near Eastern PPN B/C cultures in order to retain linearity in their models.

Here we have chosen to describe the entire process by a power law relationship between distance and age. Such a relationship entails a changing rate of spread: the dispersing wavefront starts by moving very slowly and then speeds up. This assumption is confirmed *a priori* by looking at the scatterplot of age vs distance from the oldest site in the database and *a posteriori* by looking at the model scatterplots, which clearly exhibit a power law behaviour. With this in mind we have opted to do log-log regressions. This entailed calculating the natural logarithm of both the age and distance estimates for each site and model and conduct a linear regression on these figures. When the resulting regressed line is converted back into untransformed space it produces a power law curve of best fit to the data, as will be seen in the results section.

We have also chosen to continue as in previous studies using a quantile regression [57] to model the relationship between early rice dates per site (as the dependent variable) and each site’s distance from a proposed origin of domestication (the covariate). Whereas ordinary least squares regression approximates the conditional mean of the dataset, quantile regression approximates a regression for a given quantile of the dependent variable (with the median being the 50^th^-percentile). Given that an archaeological dataset such as the present one is bound to include sites and phases, which do not correspond to the first arrival of the dispersing element being studied, regressing to the conditional mean does not always yield the most useful or valid relationship, particularly when used for inference (see debate in [58] and [41]). Data filtering or weighted regression can be used to minimize the impact of data-points that do not correspond to local first arrivals (eg [46,47]) but quantile regression provides a reassuringly robust alternative as it can approximate a low quantile without the need to choose a sub-set of the data, as in the filtering approach, or choosing an *ad hoc* weight function. Throughout this paper we have therefore used quantile regressions to the 10^th^-percentile.

### Identifying the dispersal origin

The methodology described so far allows for the regression modelling of the spread of rice in Asia based on the empirical evidence compiled in the Rice Archaeological Database *and* assuming a particular origin for the spread. This is because any measure of distance, whether a simple geodesic distances or a cost distance weighted for landscape frictions, requires a starting point—in our case representing a dispersal origin. The identification of this origin can in pratical terms be construed as an optimization problem where a given ‘index of model fitness’ is maximised when the appropriate origin is identified. Among different possible dispersal origins the one that ‘best-fits’ the empirical data is the one that should be chosen. Coefficients of correlation between radiocarbon dates and an estimate of travelled distance, are popular fitness indices for this purpose (eg [38,40,59]). The underlying logic is that stronger correlations will be found when the best dispersal origin is chosen and, therefore, one can compare different hypotheses by looking at the correlation coefficients they yield. This assumption was, as far as the authors are aware, unchecked. The rise in computational power allows for millions of random simulations to be run—the Monte Carlo method (eg [60])—which can be used to test certain methodological assumptions. These reveal the correlation coefficient to be of limited value in the identification of the source of dispersal.

A scenario was simulated wherein a dataset of 500 points was randomly constructed based on a known dispersal rule (i.e. known origin point and dispersal speed). To this underlying law, stochastic noise taken from a Rayleigh distribution of varying parameter was added. A Rayleigh distribution for the errors in age and distance ensures that the quantities used in the regression (the observables) are always underestimates of the actual points in space and time, mimicking an archaeological database where the distance travelled is always underestimated, and the radiocarbon age is always later than the actual time of first arrival (see also [41]). The Rayleigh parameter was allowed to vary in the range 0.001 to 1 over fifty equally spaced values, and resulting picks were subtracted from the underlying values, so that a noise parameter of 0.001 meant that the mode is very close to that predicted by the underlying dispersal rule, whereas a parameter of 1 meant that the noise was of the order of the value predicted by the dispersal rule. For each set of noise parameters the simulations were run for a thousand iterations. In each iteration, a grid of 121 equally spaced points (i.e. an 11x11 grid) was overlaid on the simulated data and all these points were tested as potential sources for dispersal. This involved finding the distance between each of the five hundred data-points and each of the gridpoints, then calculating the values of several fitness indices based on said distances and the simulated age of the data-points. The gridpoint that maximized each index, for each iteration, was selected and stored.

This was done for both a linear and a log-log dispersal, and involving some 605 million simulations overall. The fitness indices selected for comparison were taken from the statistical/modelling literature, namely: Pearson’s correlation coefficient *r*, Spearman’s rank correlation coefficient *ρ*, the adjusted coefficient of determination *R* ^*2*^_*adj*_, and Akaike’s Information Criterion (AIC). The first two indices are traditional correlation coefficients, whereas the latter two have been widely used for regression model selection (eg [61]). Given our use of quantile regression above and previous concerns with the validity of ordinary least squares (OLS) regression of archaeological datasets [41,58] we have calculated *R* ^*2*^_*adj*_ for OLS regression alone, since there are issues with its quantile regression analogue [62], whereas AIC was calculated for both the OLS and quantile regressions.

**Fig 3:**
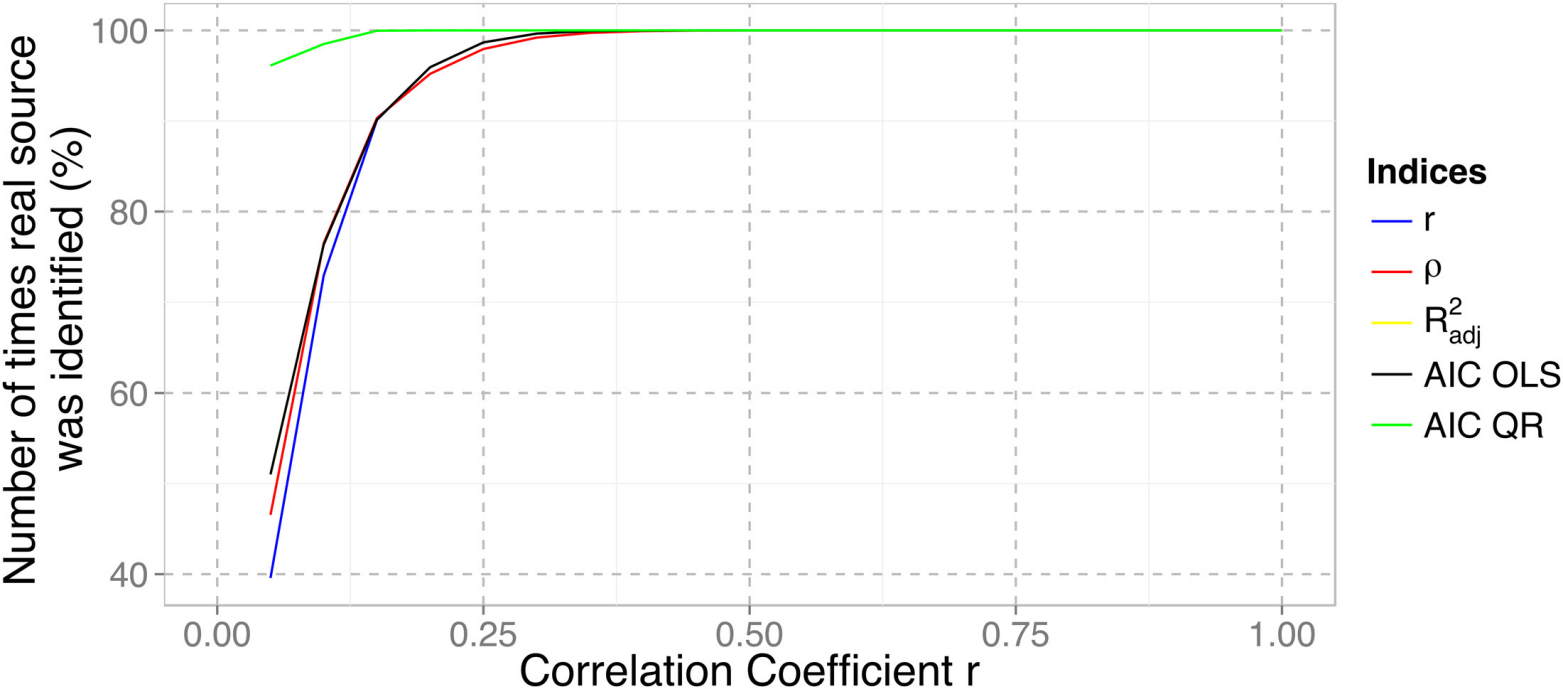
Number of times the real dispersal source was selected by different fitness indices over a range of underlying correlation coefficients. The *R* ^*2*^_*adj*_ curve (in yellow) is exactly the same, and therefore hidden behind, the AIC OLS curve (in black).

A comparison of the efficiency of each index, where the frequency with which each index picked the real source, is shown, for varying levels of noise in the dataset (represented by the correlation coefficient in the horizontal axis), in Fig 3. A correlation coefficient close to zero means that the dataset has high levels of noise and is heteroscedastic. This figure is in every way similar for both the linear and log-log/power law dispersal scenarios.

The Monte Carlo simulations reveal that the use of correlation coefficients, as well as ordinary least squares goodness-of-fit measures, are unreliable model selection indices below correlations of about 0.38. Given that archaeological datasets for continental-scale dispersals have yielded correlations in the range 0.5–0.8 (eg. [38,39,40,47]) this suggests that the choice of index is a minor concern for the identification of the geographical origins of such large-scale processes. However, lower correlation coefficients can be found on more regional datasets (e.g. [63]) and, in such cases, our simulations indicate that the fitness index must be carefully chosen.

In fact, the quantile regression AIC index seems ideal across a vast range of possibilities as it correctly identifies the true source of dispersal 96% of the time for correlations as low as 0.05. Given that using AIC with OLS regression is only marginally better than using the correlation coefficients, and exactly the same as using an *R* ^*2*^_*adj*_, we believe that this result is, at least partially, due to the use of quantile regression which, as detailed above, gives more weight to the earliest sub-set of the data. Regardless, AIC does a better job at retrieving the underlying dispersal rule and, therefore, more accurately recovers the origin of the dispersal. Moreover, the Akaike Information Criterion has been widely applied for model selection and inference, has solid foundation in both information theory and likelihood statistics [64,65] and, combined with its robust behaviour in the above Monte Carlo simulations, this make it ideally suited for our purposes and we will use it throughout, not only to find the origin area that best fits the Rice Archaeological Database, but also when quantitatively comparing this with other literature-based models (see below). Both above and below we have used the small sample corrected version for the AIC, often denominated AICc, but, for simplicity, we follow Burnham and Anderson [65] in simply naming it AIC.

### Unconstrained search for the best dispersal origin

To identify the dispersal origin(s) that best fits the archaeological dataset, an unconstrained search algorithm was used. The simplest implementation of this idea on a spatially-explicit domain is to lay out an equally spaced grid of points that will be tested as potential dispersal origins, as in the Monte Carlo simulation above. Fitness values are then calculated for each of these gridpoints as if they were the origin of dispersal. The gridpoints yielding the highest fitness will be the nearest to the dispersal origin that best-fits the entire archaeological dataset. An interpolated surface can then be constructed from this grid in order to highlight not a single gridpoint, but the area most likely to contain the dispersal origin. We choose to adopt this coarser grid search rather than use the original grid provided by the raster map shown in Fig 2 for two reasons: Firstly, in order to minimize computational time, one needs to keep the number of gridpoints to test as low as possible. The grid is therefore kept sparse, meaning that a gridpoint identified as a high fitness origin might still be far from the actual dispersal origin. And secondly, the nature of the problem, as well as the empirical data and employed statistical methodology are such that pinpointing with certainty a single origin point is highly unlikely and doubtfully meaningful. Instead, with an appropriate cut-off value, this interpolation approach can highlight a region that yields high fitness values, hopefully small enough to allow an archaeological interpretation.

This methodology has been used in both archaeology and genetics to identify the origins of the Neolithic dispersal in Europe using radiocarbon dates (eg [37,59]), and the origins of modern humans within Africa, using empirical measures of genetic diversity, such as the LD and F_st_ statistics (eg [40]), respectively. More complex variations have also been developed in order to identify the set of origin locations that maximise not one but two independent correlation coefficients, one archaeological and the other stemming from genetics data [49]. Whereas for other fitness indices, particularly the correlation coefficents or adjusted *R* ^*2*^, one would have to either choose the point with the highest value or arbitrarily choose a cut-off value in order to identify an area, an added advantage of using the Akaike Information Criterion here is that there is a natural cut-off value which one can use to highlight the best origin area. Burnham and Anderson ([65]: 70–71) demonstrated that AIC values four units higher than the lowest have very little empirical support in favour of them. Despite Anderson’s later cautious take ([66]: 89–91), this cut-off figure follows naturally from the Method of Support of Edwards [67] where a cut-off of 2 for the support, or log-likelihood, is the analogue of two standard deviations. By definition, this would yield a cut-off of 4 for the AIC.

**Fig 4:**
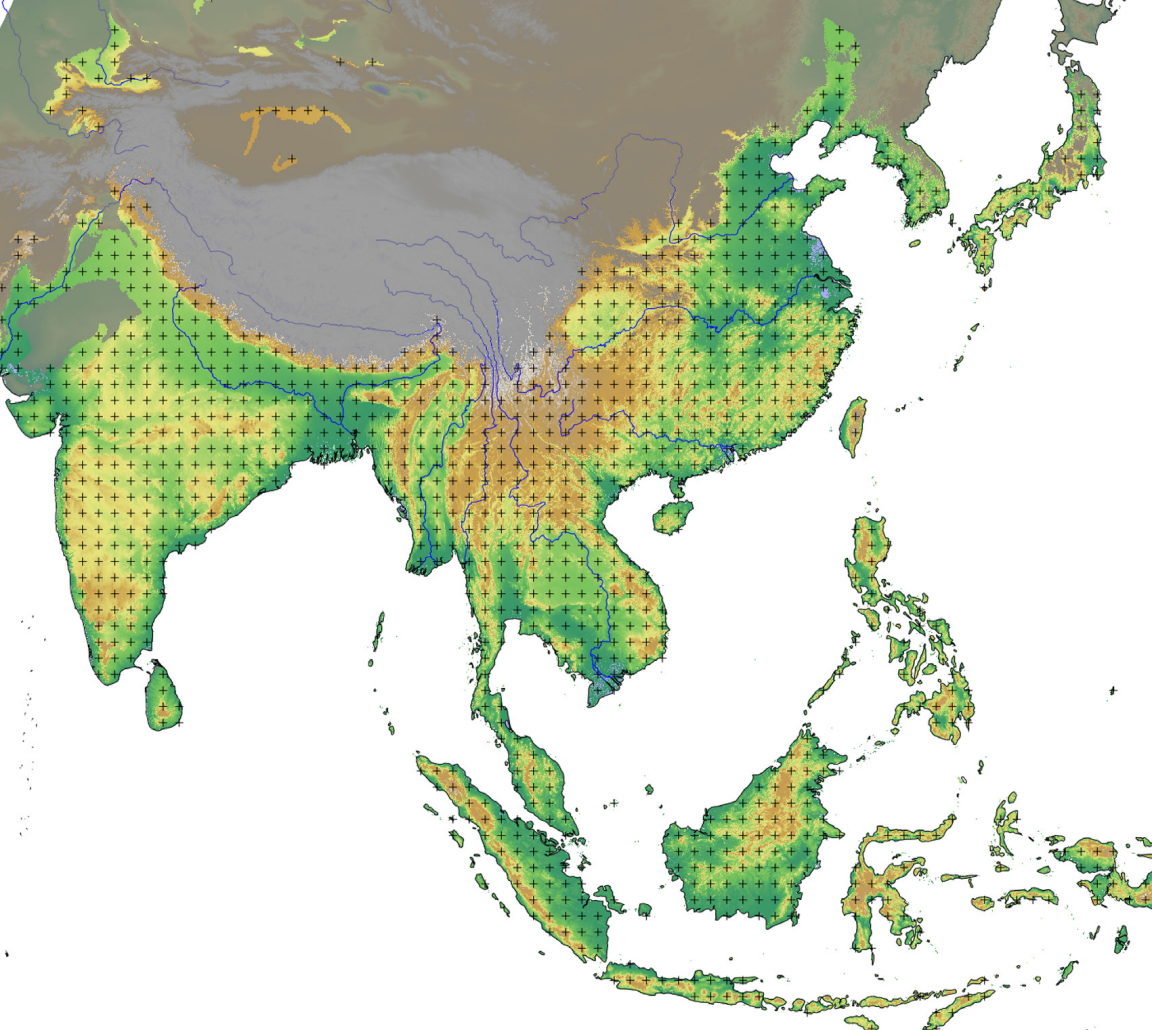
Map of the domain showing the gridpoints that were used in the unconstrained search for the best dispersal origin. The region not included in the domain is grey-shaded. For reference, the background layer includes elevation data (source: USGS Shuttle Radar Topography Mission data), as well as major rivers (source: ESRI World Major Rivers).

For this project we have created a grid of points each 100 km apart, and have masked out any grid points located outside the modelling domain defined above. This results in a total of 1275 gridpoints that act as hypothetical points of origin for the dispersal of rice (Fig 4). After obtaining the AIC values for each gridpoint, we have then used ordinary kriging (as fitted by maximum likelihood; [68]) to map them back onto the finer entire spatial domain. We then subtracted the lowest AIC value (the best origin point) from this in order to obtain Δ = AIC - AIC_min_ and thereby rescale the map.

### Comparison with literature-based models

**Fig 5:**
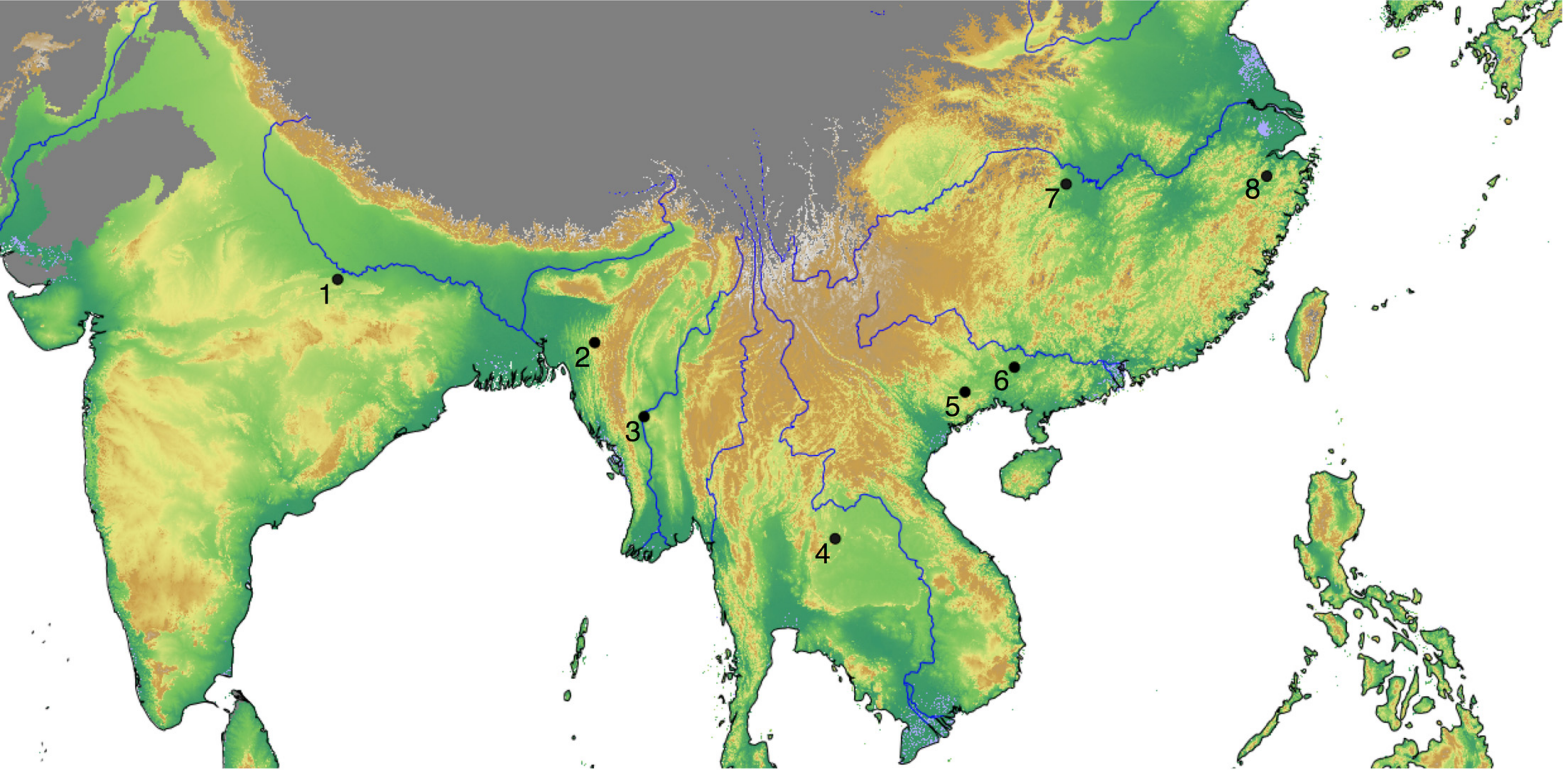
Locations of the origins used in the tested literature models. Specific archaeological sites: 1—Mahagara, 4—Non Nok Tha, 7- Pengtoushan, 8—Shangshan; general areas: 2—Assam, 3—Burma, 5—Northern Vietnam, 6—Pearl river delta. For reference, there is a background layer containing elevation data (source: USGS Shuttle Radar Topography Mission data) and the major rivers (source: ESRI World Major Rivers).

Having identified the dispersal origin that best fits the Rice Archaeological Database, one can compare the resulting model with those stemming from the literature, based on a variety of data stemming from the fields of archaeology, genetics and/or linguistics. To model such hypotheses we have chosen origin points close to the regions/sites indicated by each author(s), estimated cost distances to each archaeological site as above, executed a quantile regression of the data, and calculated the AIC and other indices that can be quantitatively compared to the results of our unconstrained search algorithm. The models used for comparison are listed (Table 1), with their imposed origin(s) mapped (Fig 5).

**Table 1:** Literature-based models tested in this paper, with their dispersal origins (numbers refer to Fig 5) and references.

Model	Dispersal Origin(s)	References
**L1**	Ganges (1), Burma (3) and northern Vietnam (5)	Chang [5]
**L2**	Ganges(1), N Thailand (4) and lower Yangtze (8)	Oka [4]
**L3**	Middle Yangtze (7) and northern Bay of Bengal littoral (2)	van Driem [27]; Hazarika [29]
**L4**	Pearl river delta (6)	Huang et al [10]
**L5**	Middle Yangtze (7)	Pei [14]; Crawford and Shen [30]; Toyama [31]; Higham [32]
**L6**	Lower Yangtze (8)	Jiang and Liu [13]; Bellwood [33]
**L7**	Middle and Lower Yangtze (7 and 8)	Fuller and Qin [17]; Fuller et al [20,30]

Some of these hypotheses involve more than one centre of innovation, implying more than one dispersal process and hence extra parameters in their modelling. The two key parameters are the rates of spread and starting time offset of each extra dispersal process [51]. Since all these literature-based hypotheses suggest similar, or the same, dates for the identified centres of innovation we have forced all modelled dispersal processes to start at the same time. The remaining parameters, the dispersal speeds, were normalized to one of the processes, so that the parameters to be estimated are reduced speed ratios: how much faster, or slower, the second and/or third independent dispersal process is with respect to the first one, following the order given in Table 1. These have been estimated by maximizing the log-likelihood (via unified optimization, see [69]), where we have implemented an upper bound of 20 and a lower of 0.01 for the parameter values.

Given the inclusion of extra parameters in some of the models one needs to be careful about our model selection approach, as models with more parameters will almost always fit the data better. Nevertheless, the AIC is still a reliable index as it penalises less parsimonious models, thereby preferring a model that not only best-fits but does not do so at the cost of adding extra parameters [65]. The best model is the one with the lowest AIC value and it is thefore useful to calculate a Δ value by subtracting this value from the AIC values of all models (as we have also done for the unconstrained search, see above). This can then be used to calculate *L(g*_*i*_ *|x)*, the likelihood of the model given the data, and *w*_*i*_, the Akaike weights or model probabilities, which provide measures of support for the different models ([65]: 74–80).

All rasters and map outputs were generated in GRASS GIS [69]. The modified Fast Marching implementation was coded in MATLAB (version R2014b). All other modelling and output generation was done in R [70], in particular: quantile regression was done using the *quantreg* package [71], parameter estimation was done using the unified optimization algorithm of package *optimx* [72], kriging was done using the *geoR* package [73], and the interaction between MATLAB and R was managed using the *R*.*matlab* package [74].

## Results

**Fig 6:**
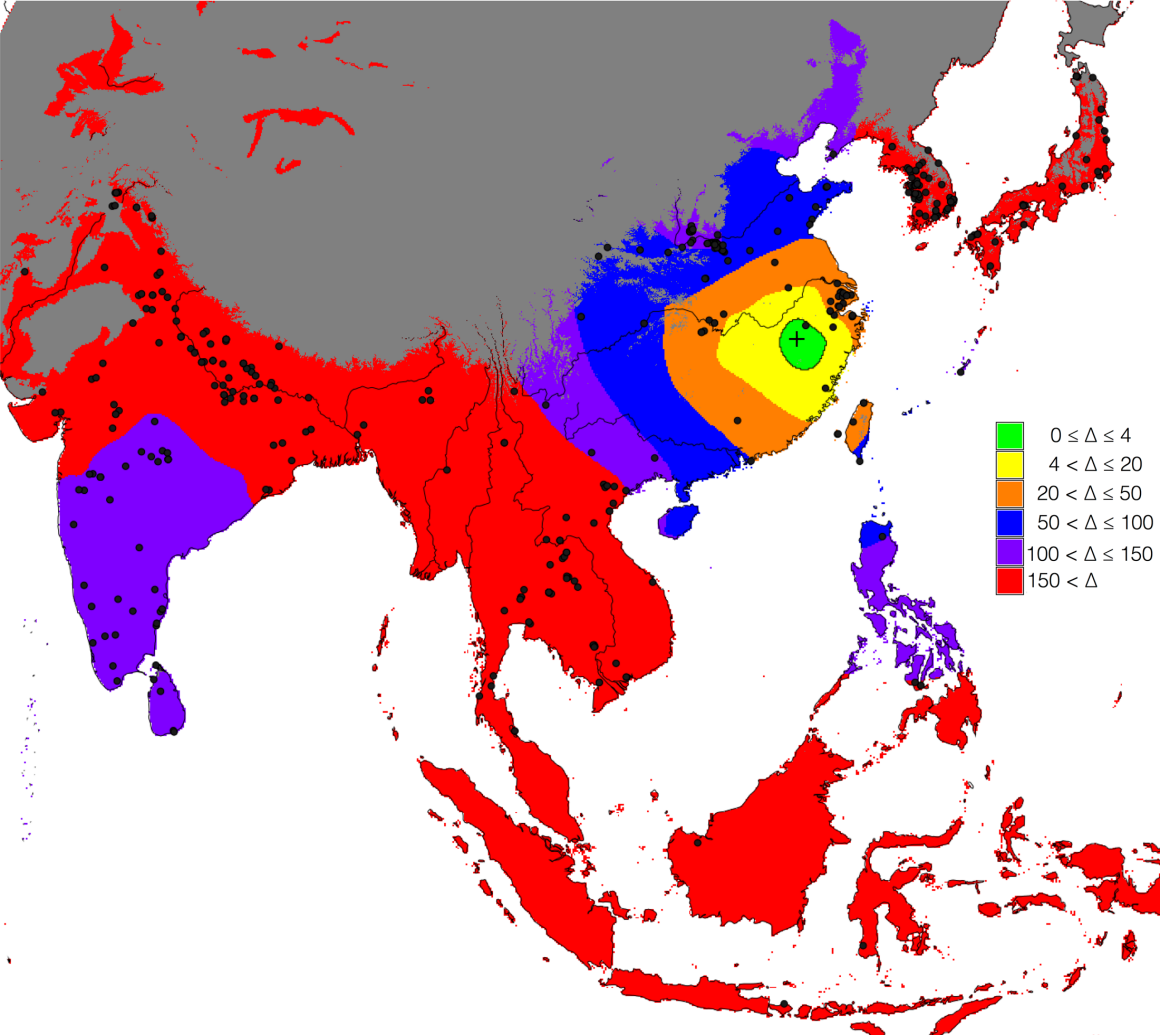
Interpolated map of ΔAIC values obtained from the results of the unconstrained search algorithm. The gridpoint with lowest AIC is marked by the cross. The black dots mark the sites with cultivated rice in the Rice Archaeological Database. Major rivers are also shown, for reference (source: ESRI World Major Rivers).

The unconstrained search algorithm identified a single region as the most likely to contain the dispersal origins for rice farming. Fig 6 shows the resulting map of Δ values. The contour line shows Δ values of 4, therefore delimiting the area with the most evidence in support of it being the dispersal origin. The best AIC value, corresponding to a Δ of zero in this figure (marked by a cross in the figure), is given in Table 2, where it can be compared with that of the other, literature-based models.

**Table 2:** Results for all considered models. Models numbered L# are literature-based, whereas model Unc is the result of the unconstrained search. Estimated parameters are speed ratios with relation to the first process, except for model Unc where the coordinates of the best-fitting source are given instead. N stands for sample size, K for the number of parameters of each model.

Model	Estimated Parameters	N	K	Log-likelihood	AIC	Δ	*L(g*_*i*_ *|x)* and *w*_*i*_
**L7**	**{1, 0.78}**	**330**	**4**	**-111.5053**	**231.1338**	**0**	**1**
**Unc**	{29.05°N, 117.65°E}	330	3	-142.7261	291.5259	60.3921	8 x 10^-14^
**L6**	{1}	330	3	-148.8051	303.6839	72.5501	2 x 10^-16^
**L2**	{1, 0.014, 20}	330	5	-147.1427	304.4705	73.3367	1 x 10^-16^
**L5**	{1}	330	3	-161.6986	329.4707	98.3369	4 x 10^-22^
**L3**	{1, 19.4}	330	4	-161.6662	331.4556	100.3218	2 x 10^-22^
**L4**	{1}	330	3	-189.0557	384.185	153.0512	6 x 10^-34^
**L1**	{1, 0.014, 0.01}	330	5	-228.5263	467.2378	236.104	5 x 10^-52^

**Fig 7:**
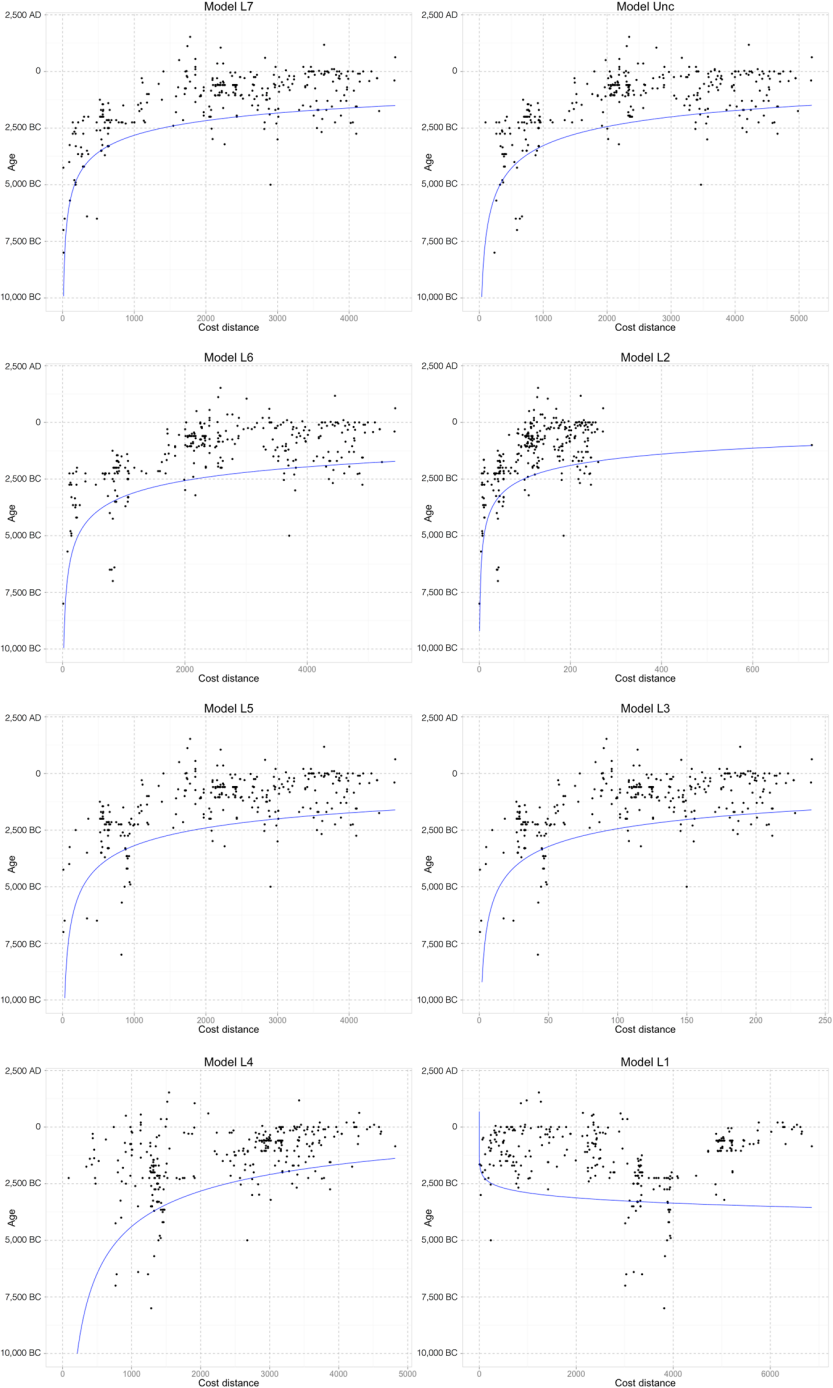
Scatterplots of age with cost distance for all models considered. The black dots represent records in the Rice Archaeological Database whereas the blue line represents the best-fitting log-log quantile regressed line for each model.

Table 2 shows the AIC values obtained for all hypotheses considered, ordered by how well they perform. Also included are all quantities used to calculate the AIC values, as well as the Δ values and model likelihood, which provide a measure of support. Scatterplots for each of the models are provided (Fig 7). The black dots correspond to data on the Rice Archaeological Database, whereas the red curve corresponds to the maximum likelihood estimated curve for each hypothesis, given the data.

## Discussion

Our methodology has identified a wide area in China as the dispersal origin that best fits the Rice Archaeological Database. This area lies in the region with the oldest known archaeological evidence for rice domestication in Asia and this demonstrates that identifying the wider Yangtze valley as the oldest centre of innovation for rice farming is not simply a fluke of archaeological sampling, but that it is supported by the entirety of available archaeological evidence for the presence of cultivated rice in Asia.

The unconstrained search algorithm identified an area between the lower and middle Yangtze regions as the most likely source for the dispersal of rice. The gridpoint with lowest AIC value, located at 29.05° N 117.65° E, lies in the northeast of the Jiangxi province, a region with little archaeobotanical evidence for rice cultivation. However, the algorithm was inherently looking for the best-fitting *single* source and could not identify multiple origin scenarios. Under such constraints, and in the presence of several simultaneous origins, such algorithms will always go for the spatial equivalent of an average: they highlight an area in-between the different real origins. They can even yield lower fits to the individual origin regions when compared to the spatially averaged area. Such mathematical artefacts highlight the importance of balancing purely statistical approaches, such as the unconstrained search approach above, with the explicit modelling of archaeologically-informed hypotheses.

**Fig 8:**
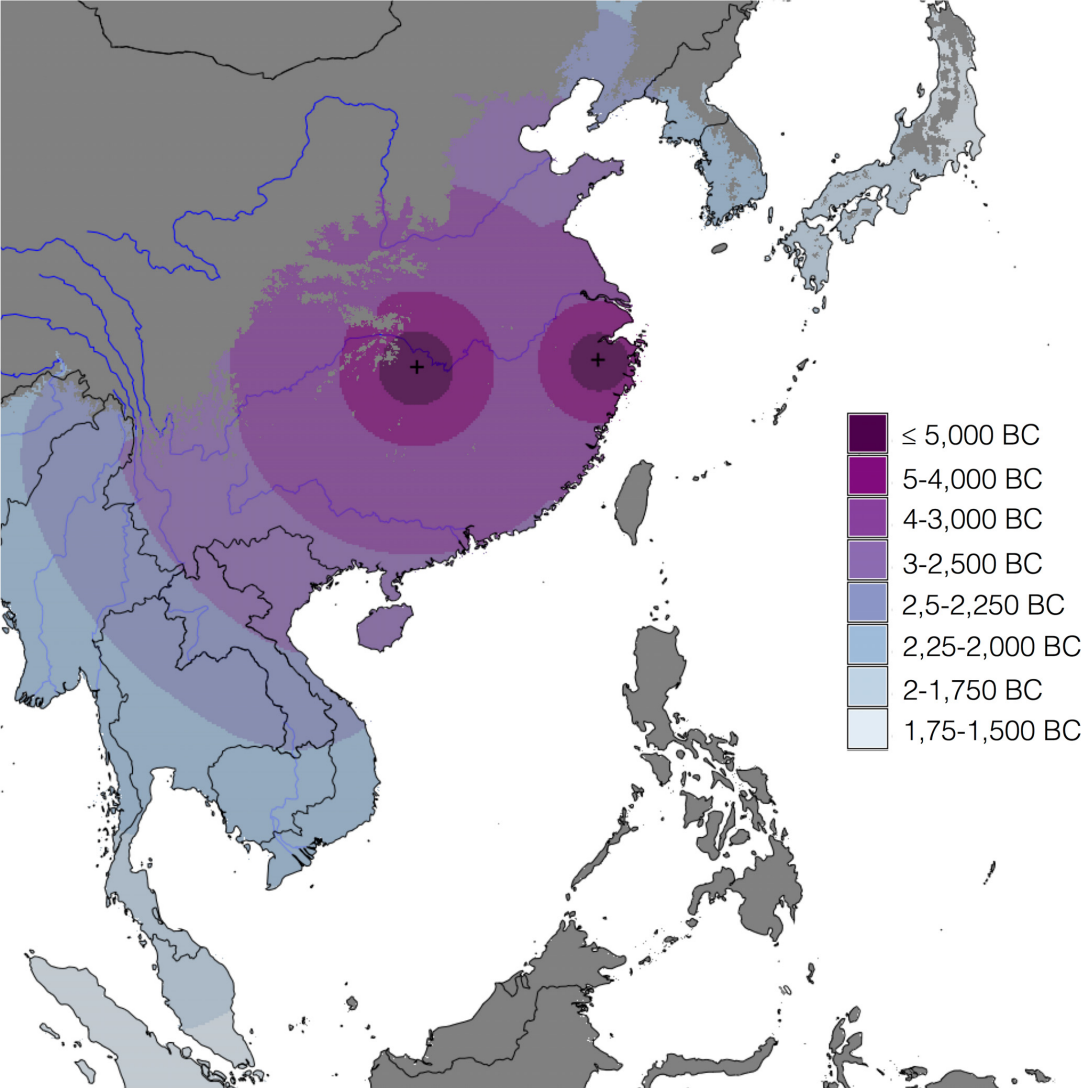
Predicted arrival times of cultivated rice in eastern and southeastern Asia, based on best-fitting model L7. Areas in dark grey were not included in the modelling framework (see main text). Major rivers (source: ESRI World Major Rivers) and country borders shown for reference.

Because of this, and despite being the best-fitting *single* origin model, and the second best overall, the model chosen by our unconstrained search algorithm is largely outperformed by model L7, which postulates two independent origins in the Yangtze basin (Fig 8). The dual Yangtze model outperforms all others to such an extent that all the weight of evidence is in support of it, as shown by the calculated model likelihoods and Akaike weights. In effect, the difference in log-likelihood between model L7 and the second best supported literature model, the Lower Yangtze one (L6), corresponds to the random draw of over 125 million white balls out of an urn and asking whether this is sufficient evidence that the urn contains only white balls, versus containing an equal amount of white and black balls [75]. The evidence is therefore overwhelmingly in favour of model L7 over all other models.

Beyond this useful result, it is further instructive to look at the estimated parameter values for the multi-centre hypotheses, given in Table 2. Reminding ourselves that these parameters are the ratios of the diffusion rates of the different processes (always with respect to the first one as given in Table 1), it becomes clear that with the exception of the double Yangtze model (L7) where both processes have comparable rates of spread (a ratio of 0.78), all others provide better fits to the data when one process dominates the others, as indicated by extreme ratios of 0.01, on the one hand, and 20, on the other. Namely, models L2 and L3 have more explanatory power when they are closer to models L6 and L5, respectively (as evidenced by the AIC values), that is when their lower and middle Yangtze processes, respectively, are fast enough to spread through the available domain before the other processes disperse far from their origins. In other words, models L2 and L3 are only good (in relative terms, as we have seen) when they mimic models L6 and L5. Furthermore, model L1, which doesn’t consider the Yangtze valley as a centre of innovation at all, is dominated by a diffusion process starting in the Ganges. However, this results in the worst fitting model of our set, with a Δ of about 236.

Even though it might be argued that we have not chosen the best possible origin points to represent the literature-based models, the behaviour of models L1-L3 just discussed, combined with the highlighting of the Yangtze basin by the unconstrained search algorithm and the overwhelming evidence in favour of model L7, indicate that the available archaeological evidence for the presence of cultivated rice across Asia is best described by a narrative involving two independent but contemporaneous centres of cultivation, one in the Lower Yangtze and the other in the Middle Yangtze.

**Fig 9:**
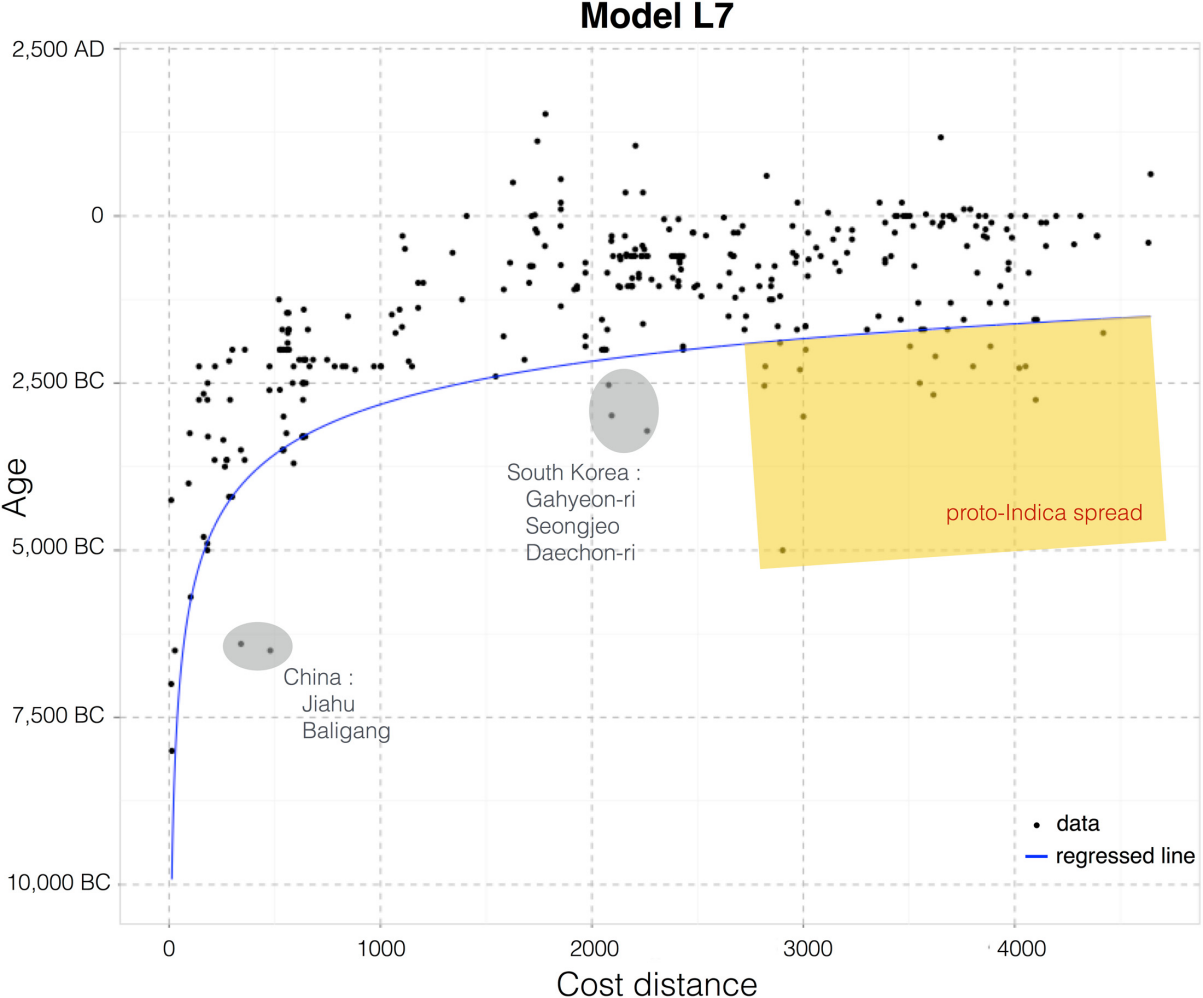
Scatterplot of the dataset for the best-fitting model L7. The quantile interpolation of the 10^th^-percentile of the data (blue line) shows that the distribution follows a power law rule. Sites earlier than the model are circled and identified (see the text for related discussion).

Finally, one can look at the scatterplot for the best-fitting model in search of outliers and different signals that might help direct future archaeological sampling as well as identify biogeographical features that impacted the spread of rice outside of the Yangtze valley (Fig 9). Since we are interested in origins and the dispersal of rice farming in Asia we can define outliers as those sites that are older than predicted by the best-fitting model, given by Eq 1 below. There are only a handful of these in temperate regions: Jiahu and Baligang in the Henan province of China, and Gahyeon-ri, Seongjeo and Daechon-ri in South Korea (grey shaded in Fig 9). The first two might indicate a third early centre of innovation between the Yangtze and Yellow rivers or trade between this region and the Yangtze valley. Whereas the Korean sites might indicate that rice arrived by crossing the Yellow sea, something that was not included in any of the above models and that would shorten the cost distances between these sites and the origins of the dispersal. The reported date of these sites may also be spurious, as these are not directly-dated rice remains. Other critical reviews, for example, have suggested that rice may be intrusive and younger at Daechon-ri and mis-dated at the other sites [76]. In either case, they are only a handful of sites in each region and further archaeological sampling is necessary in order to test and further refine these ideas. It should also be noted that the arrival time for rice in Japan, predicted by the model is significantly earlier (ca. 2,000–1,500 BC) than the generally accepted terminal or post-Jomon arrival (ca. 1,000–800 BC) [35,77]. This suggests that factors of cultural choice may have resisted rice in the Japanese islands, despite millennia of plant management and some domestications by Jomon societies [77]. In tropical regions, namely in northern India, we find several sites with possibly cultivated rice that can predate model L7 by more than a millennium (as much as 3,000 years for the case of Lahuradewa). This might correspond to an independent episode of domestication of proto-Indica rice and its subsequent spread up the Ganges valley [17,34]. However, the Early Holocene evidence from Lahuradewa in India, remains unclear as to whether this material was actually cultivated and was part of an independent domestication trajectory [27,78], and further work is needed.

$$age=20346.7404\;\cdot \;distance^{-0.210026}$$ (1)

## Conclusion

In this paper we present results of the spatial modelling for the origins of rice cultivation that lead to the original domestication or domestications of *Oryza sativa* subsp. *japonica*. This is based on an extensive database of empirical archaeological evidence for rice, which represents the fossil record of the relationship between past human societies and this important crop. This modelling approach provides robust, explicit hypotheses of the regions or region in which the earliest evidence for rice should be sought. This agrees with many archaeologists, who have recently focused on the Middle and Lower Yangtze basin (e.g. [8,18,35,79]). There is no basis on the present evidence to privilege the Lower Yangtze (Zhejiang province) or Middle Yangtze (Hunan province) as a more likely source region of a singular rice domestication episode. Instead, multiple, distinct domestication episodes seems the most plausible hypothesis in the current state of our evidence. Indeed cultural differences between the Neolithic traditions of the Lower and Middle Yangtze (e.g. [80]), including the earliest preserved field systems [17], argues against diffusion of rice cultivation between the two centres of innovation.

The origins of rice remains of interest to archaeologists, historical linguists and plant geneticists, but there is no consensus of where this was located. Genetic evidence has been limited by the modern distribution of wild populations, which is likely a poor reflection of where wild rice was found in the early Holocene when rice cultivation began, meaning that those wild populations involved in domestication have been extirpated. The possible, but always highly indirect contribution of linguistic evidence likely suffers from a similar problem, in that we are limited by extant, or recently written down, languages, and therefore extinct language groups that may have played a role in domestication cannot be easily taken into account. Indeed in the Yangtze basin language shift to Sinitic languages is known to have occurred over the course of the Bronze and Iron Ages under the political influence of the central Chinese state, and thus we are left with only unprovable hypotheses of which modern or extinct language families might have been present in the region during the Neolithic (e.g. [34]). Archaeobotanical evidence has the advantage of being fixed in past time and in space, although the gaps between archaeobotanical datapoints must be filled in by inference. We have demonstrated in this study how spatial modelling and the fast-marching method to generating cost-surfaces for diffusion can effectively fill in gaps in the archaeobotanical record and produce explicit hypotheses that can help to direct further problem-oriented archaeological sampling.

## Supporting Information

S1 Map Rice Archaeological Database v2. (KMZ)

## References

[1] Bray F. The Rice Economies: Technology and Development in Asian Societies. Oxford: Blackwell; 1986.

[2] Gurou P. Riz et civilisation. Paris: Fayard; 1984.

[3] Scott JC. The Art of Not Being Governed: An Anarchist History of Upland Southeast Asia. New Haven: Yale University Press; 2009.

[4] Oka HI. Origins of Cultivated Rice. Oxford, New York and Tokyo: Japan Scientific Societies Press and Elsevier; 1988.

[5] Chang TT. Domestication and the spread of the cultivated rices In: Harris DR and Hillman GC, editors. Foraging and Farming: The Evolution of Plant Exploitation. London: Unwin Hyman; 1989 pp. 408–417.

[6] Yasuda Y. Origins of pottery and agriculture in East Asia In: Yasuda Y, editor. The Origins of Pottery and Agriculture. New Delhi: Lustre Press/Roli Books; 2002 pp. 119–142.

[7] Vaughn DA, Lu B, Tomooka N. Was Asia rice (Oryza sativa) domesticaded more than once? Rice 2008; 1: 16–24. 10.1007/s12284-008-9000-0

[8] Zhang C, Hung HC. The emergence of agriculture in southern China. Antiquity 2010; 84(323): 11–25. 10.1017/S0003598X00099737

[9] Londo JP, Chiang Y, Hung K, Chiang T, Schaal BA. Phylogeography of Asian wild rice, Oryza rufipogon, reveals multiple independent domestications of cultivated rice, Oryza sativa. Proceedings of the National Academy of Sciences 2006; 103: 9578–9583. 10.1073/pnas.0603152103 PMC1480449 16766658

[10] Huang X, Kurata N, Wei X, Wang Z-X, Wang A, Zhao Q, et al A map of rice genome variation reveals the origin of cultivated rice. Nature 2012; 490: 497–501. 10.1038/nature11532 23034647 PMC7518720

[11] Lu H, Liu Z, Wu N, Berné S, Saito Y, Liu B, et al Rice domestication and climatic change: phytolith evidence from East China. Boreas: An International Journal of Quaternary Research 2006; 31(4): 378–385. 10.1111/j.1502-3885.2002.tb01081.x

[12] Zhao Z. The Middle Yangtze region in China is one place where rice was domesticated: phytolith evidence from the Diaotonghuan Cave, Northern Jiangxi. Antiquity 1998; 72: 885–897.

[13] Jiang L, Liu L. New evidence for the origins of sedentism and rice domestication in the Lower Yangtze River, China. Antiquity 2006; 80: 355–361. 10.1017/S0003598X00093674

[14] Pei A. Notes on new advancements and revelations in the agricultural archaeology of early rice domestication in the Dongting Lake region. Antiquity 1998; 72: 878–85.

[15] Fuller DQ, Harvey E, Qin L. Presumed domestication? Evidence for wild rice cultivation and domestication in the fifth millennium BC of the Lower Yangtze region. Antiquity 2007; 81: 316–331. 10.1017/S0003598X0009520X

[16] Fuller DQ, Qin L, Zheng Y, Zhao Z, Chen X, Hosoya LA, et al The Domestication Process and Domestication Rate in Rice: Spikelet bases from the Lower Yangtze. *Science* 2009; 323: 1607–1610. 10.1126/science.1166605 19299619

[17] Fuller DQ, Qin L. Water management and labour in the origins and dispersal of Asian rice. World Archaeology 2009; 41(1): 88–111. 10.1080/00438240802668321

[18] Crawford GW. Early rice exploitation in the lower Yangzi valley: What are we missing?. The Holocene 2012; 22(6): 613–621. 10.1177/0959683611424177

[19] Gross BL, Zhao Z. Archaeological and genetic insights into the origins of domesticated rice. Proceedings of the National Academy of Sciences 2014; 111(17): 6190–6197. 10.1073/pnas.1308942110 PMC4035933 24753573

[20] Fuller DQ, Denham T, Arroyo-Kalin M, Lucas L, Stevens CJ, Qin L, et al Convergent evolution and parallelism in plant domestication revealed by an expanding archaeological record. Proceedings of the National Academy of Sciences 2014; 111(17): 6147–6152. 10.1073/pnas.1308937110 PMC4035951 24753577

[21] Harris DR, Fuller DQ. Agriculture: Definition and Overview In Smith C, editor. Encyclopedia of Global Archaeology. New York: Springer; 2014 pp 104–113.

[22] Higham C. Languages and farming dispersals: Austroasiatic languages and rice cultivation In: Bellwood P and Renfrew C, editors. Examining the farming/language dispersal hypothesis. Cambridge: McDonald Institute for Archaeological Research; 2003 pp. 223–232.

[23] Bellwood P. First Farmers. Oxford: Blackwell; 2005.

[24] Blench RM. From the mountains to the valleys: understanding ethnolinguistic geography in Southeast Asia In: Blench RM, Sagart L, Sanchez-Mazas A, editors. Perspectives in the phylogeny of East Asian languages. London: Curzon; 2005 pp. 31–50.

[25] Sagart L. The expansion of Setaria farmers in East Asia: a linguistic and archaeological model In: Sanchez-Mazas A, Blench RM, Ross M, Lin M, Pejros I, editors. Human migrations in continental East Asia and Taiwan: matching archaeology, linguistics and genetics. London: Taylor & Francis; 2008 pp. 133–157.

[26] Sagart L. How many independent rice vocabularies in Asia? Rice 2011; 4(3–4): 121–133. 10.1007/s12284-011-9077-8

[27] van Driem G. Neolithic correlates of ancient Tibeto-Burman migrations In: Blench R and Spriggs M, editors. Archaeology and Language II: Archaeological Data and Linguistic Hypotheses; 1998 pp. 67–102.

[28] van Driem G. The ethnolinguistic identity of the domesticators of Asian rice. Comptes Rendus Palevol 2012; 11(2–3): 117–132. 10.1016/j.crpv.2011.07.004

[29] Hazarika M. Neolithic Culture of Northeast India: A Recent Perspective on the Origins of Pottery and Agriculture. Ancient Asia 2006; 1:25–44. 10.5334/aa.06104

[30] Crawford GW, Shen C. The origins of rice agriculture: recent progress in Asia. Antiquity 1998; 72(278): 858–866.

[31] Toyama S. The origin and spread of rice cultivation as seen from rice remains In: Yasuda Y, editor. The origins of pottery and agriculture. New Delhi: Lustre Press/Roli Books; 2002 pp. 263–272.

[32] Higham C. East Asian Agriculture and Its Impact In: Scarre C, editor. The Human Past. London: Thames and Hudson; 2005 pp. 234–263.

[33] Bellwood P. Prehistory of the Indo-Malaysian Archipelago. 2nd ed Honolulu: University of Hawaii Press; 1997.

[34] Fuller DQ. Pathways to Asian civilizations: Tracing the origins and spread of rice and rice cultures. Rice 2011; 4(3–4): 78–92. 10.1007/s12284-011-9078-7

[35] Fuller DQ, Sato Y-I, Castillo C, Qin L, Weisskopf AR, Kingwell-Banham EJ, et al Consilience of genetics and archaeobotany in the entangled history of rice. Archaeological and Anthropological Sciences 2010; 2 (2): 115–131. 10.1007/s12520-010-0035-y

[36] Fuller DQ, van Etten J, Manning K, Castillo C, Kingwell-Banham EJ, Weisskopf AR, et al The contribution of rice agriculture and livestock pastoralism to prehistoric methane levels An archaeological assessment. The Holocene 2011; 21: 743–759. 10.1177/0959683611398052

[37] Weisskopf A, Qin L, Ding J, Sun G, Fuller DQ. Phytoliths and rice: from wet to dry and back again in the Neolithic Lower Yangtze. Antiquity 2015; 89 (346).

[38] Pinhasi R, Fort J, Ammerman AJ. Tracing the Origin and Spread of Agriculture in Europe. PLoS Biol 2005; 3(12): e410 10.1371/journal.pbio.0030410 16292981 PMC1287502

[39] Fort J, Pujol T, Vander Linden M. Modelling the Neolithic transition in the Near East and Europe. American Antiquity 2012; 77 (2): 203–219. 10.7183/0002-7316.77.2.203

[40] Henn BM, Gignoux CR, Jobin M, Granka JM, Macpherson JM, Kidd JM, et al Hunter-gatherer genomic diversity suggests a southern African origin for modern humans. Proceedings of the National Academy of Sciences of the United States of America 2011; 108 (13): 5154–5162. 10.1073/pnas.1017511108 21383195 PMC3069156

[41] Silva F, Steele J. New methods for reconstructing geographical effects on dispersal rates and routes from large-scale radiocarbon databases. Journal of Archaeological Science 2014; 52: 609–620. 10.1016/j.jas.2014.04.021

[42] Douglas DH. Least cost path in GIS using an accumulated cost surface and slope lines. Cartographica 1994; 31(3): 27–51. 10.3138/D327-0323-2JUT-016M

[43] Connolly J, Lake M. Geographic Information Systems in Archaeology. Cambridge: Cambridge University Press; 2006.

[44] Saerens M, Achbany Y, Fouss F, Yen L. Randomized shorthest-path problems: two related models. Neural Computation 2009; 21: 2363–2404. 10.1162/neco.2009.11-07-643 19323635

[45] de Smith MJ, Goodchild MF, Longley PA. Geospatial Analysis A Comprehensive Guide to Principles, Techniques and Software Tools. 2nd ed Leicester: Troubador Publishing; 2007.

[46] Silva F, Steele J, Gibbs K, Jordan P. Modeling spatial innovation diffusion from radiocarbon dates and regression residuals: the case of early old world pottery. Radiocarbon 2014; 56(2): 723–732. 10.2458/56.16937

[47] Russell T, Silva F, Steele J. Modelling the spread of farming in the Bantu-speaking regions of Africa: an archaeology-based phylogeography. PLoS One 2014; 9(1): e87854 10.1371/journal.pone.0087854 24498213 PMC3909244

[48] MacRae BH, Beier P. Circuit theory predicts gene flow in plant and animal populations. Proceedings of the National Adademy of Sciences 2007; 104(5): 19885–19890. 10.1073/pnas.0706568104 PMC2148392 18056641

[49] van Etten J, Hijmans RJ. (2010) A Geospatial Modelling Approach Integrating Archaeobotany and Genetics to Trace the Origin and Dispersal of Domesticated Plants. PLoS ONE 2010; 5(8): e12060 10.1371/journal.pone.0012060 20711460 PMC2920323

[50] Sethian JA. A fast marching level set method for monotonically advancing fronts. Proceedings of the National Academy Sciences 1996; 93(4): 1591–1595. 10.1073/pnas.93.4.1591 PMC39986 11607632

[51] Silva F, Steele J. Modeling boundaries between converging fronts in prehistory. Advances in Complex Systems 2012; 15(01n02): 1150005 10.1142/S0219525911003293

[52] Bonhomme R. Bases and limits to using ‘degree.day’ units. European Journal of Agronomy 2000; 13: 1–10.

[53] Guedes JDA, Butler EE. Modeling constraints on the spread of agriculture to Southwest China with thermal niche models. Quaternary International 2014; 349: 29–41. 10.1016/j.quaint.2014.08.003

[54] Guedes JDA, Lu H, Hein AM, Schmidt AH. Early evidence for the use of wheat and barley as staple crops on the margins of the Tibetan Plateau. Proceedings of the National Academy of Sciences 2015; 10.1073/pnas.1423708112 PMC4426421 25902511

[55] Hijmans RJ, Cameron SE, Parra JL, Jones PG, Jarvis A. Very high resolution interpolated climate surfaces for global land areas. International Journal of Climatology 2005; 25: 1965–1978. 10.1002/joc.1276

[56] Olson DM, Dinerstein E, Wikramanayake ED, Burgess ND, Powell GV, Underwood EC, et al Terrestrial ecoregions of the world: a new map of life on earth. BioScience 2001; 51: 933–938. 10.1641/0006-3568(2001)051[0933:TEOTWA]2.0.CO;2

[57] Koenker R. Quantile Regression. Cambridge, Cambridge University Press; 2005.

[58] Steele J. Radiocarbon dates as data: quantitative strategies for estimating colonization front speeds and event densities. Journal of Archaeological Science 2010; 37(8): 2017–2030. 10.1016/j.jas.2010.03.007

[59] Ammerman AJ, Cavali-Sforza LL. Measuring the rate of spread of early farming in Europe. Man NS 1971; 6: 674–688.

[60] Fishman GS. Monte Carlo: Concepts, Algorithms, and Applications. New York: Springer; 1995.

[61] McQuarrie ADR, Tsai C-L. Regression and Times Series Model Selection. Singapore, World Scientific Publishing Co; 1998.

[62] Koenker R, Machado JAF. Goodness of Fit and Related Inference Processes for Quantile Regression. Journal of the American Statistical Association 1999; 94(448): 1296–1310. 10.1080/01621459.1999.10473882

[63] Aubán JB, Barton CM, Gordó SP, Bergin SM. Modelling initial Neolihtic dispersal. The first agricultural groups in West Mediterranean. Ecological Modelling 2015; 307: 22–31.

[64] Akaike H. Information theory as an extension of the maximum likelihood principle In: Petrov BN and Caski F, editors. Second International Symposium on Information Theory. Budapest: Akademiai Kiado; 1973 pp.267–281.

[65] Burnham KP, Anderson DR. Model Selection and Multimodel Inference: A Practical Information-Theoretic approach. 2nd ed New York: Springer; 2002.

[66] Anderson DR. Model Based Inference in the Life Sciences: a primer on evidence. New York: Springer; 2008.

[67] Edwards AWF. Likelihood Expanded edition. Baltimore: Johns Hopkins University Press; 1992.

[68] Krige DG. A statistical approach to some basic mine valution problems on the Witwatersrand. Journal of the Chemical, Metallurgical and Mining Society of South Africa 1951; 52: 119–139.

[69] GRASS Development Team. Geographic Resources Analysis Support System (GRASS 7) Programmer's Manual. Open Source Geospatial Foundation Project; 2012. Available: http://grass.osgeo.org/programming7/.

[70] R Core Team. R: A language and environment for statistical computing. Vienna: R Foundation for Statistical Computing; 2014 Available: http://www.R-project.org/.

[71] Koenker R. quantreg: Quantile Regression. R package version 5.05. 2013 Available: http://CRAN.R-project.org/package=quantreg

[72] Nash JC, Varadhan R. Unifying Optimization Algorithms to Aid Software System Users: optimx for R. Journal of Statistical Software 2001; 43(9), 1–14. Available: http://www.jstatsoft.org/v43/i09/.

[73] Ribeiro PJ Jr, Diggle PJ. geoR: a package for geostatistical analysis. R-NEWS 2001; 1(2): 15–18.

[74] Bengtsson H. R.matlab: Read and write MAT files and call MATLAB from within R. R. pacakge version 3.2.0. Available: http://CRAN.R-project.org/package=R.matlab

[75] Royall R. Statistical Evidence: A Likelihood Paradigm. Boca Raton: CRC Press; 2000.

[76] Ahn S-M. The emergence of rice agriculture in Korea: archaeobotanical perspectives. Archaeological and Anthropological Perspectives 2010; 2: 89–98. 10.1007/s12520-010-0029-9

[77] Crawford GW. Advances in Understanding Early Agriculture in Japan. Current Anthropology 2011; 52(S4): S331–S345. 10.1086/658369

[78] Fuller DQ. Finding plant domestication in the Indian subcontinent. Current Anthropology 2011; 52(S4): S347–S362. 10.1086/658900

[79] Shelach-Lavi G. The Archaeology of Early China: From Prehistory to the Han Dynasty. Cambridge: Cambridge University Press; 2015.

[80] Makibaiyahi K. The Transformation of Farming Cultural Landscapes in the Neolithic Yangtze Area, China. Journal of World Prehistory 2014; 27(3–4): 295–307. 10.1007/s10963-014-9082-0

